# High-intensity circuit training for improving anthropometric parameters for women from low socioeconomic communities of Sikandarabad: A clinical trial

**DOI:** 10.1371/journal.pone.0275895

**Published:** 2022-10-17

**Authors:** Sana Mehmood, Amna Khan, Sumaira Farooqui, Al-Wardha Zahoor, Qurat Ul Ain Adnan, Usman Khan

**Affiliations:** Ziauddin College of Rehabilitation Sciences, Ziauddin University, Karachi, Pakistan; California State University San Marcos, UNITED STATES

## Abstract

**Background:**

An alarming trend of sustained physical inactivity has been observed among women in socioeconomically disadvantaged areas, mainly due to the lack of time and high cost of gym facilities. Although physical activity essentially contributes to disease prevention, evidence supporting time-efficient exercise on anthropometric measures is limited. This study aimed to identify the effectiveness of interval-based high-intensity circuit training (HICT) on anthropometric measures and the nature of the relationship between these measures.

**Methods:**

A single-group, quasi-experimental study was conducted in the community park of Ziauddin Hospital at Sikandarabad. Sixty women who were overweight and had sedentary lifestyles were recruited for a six-week HICT-based program conducted at 85%–95% maximum heart rate (MHR) on every alternate day. Outcome measures were assessed at baseline and at 6-weeks including anthropometric parameters (body mass index [BMI], body fat percentage [BF%], and waist-to-hip ratio [WHR]).

**Results:**

The six-week HICT-based program demonstrated a significant reduction in BMI (*p*<0.001), BF% (*p*<0.001), and WHR (*p*<0.001). Reductions in the BMI mean from 27.3±1.3 to 25.1±1.4 and BF% mean from 31.9±2.3 to 27.6±2.4 were observed following 18 sessions of HICT. The effect of age on BF% and WHR was linearly significant (*p*<0.001) with increasing age (BF%) and WHR.

**Conclusion:**

Interval-based HICT was an effective exercise regimen for improving BMI, BF%, and WHR. Furthermore, the exercise protocol was feasible and well tolerated, with no reported adverse events, and it could be easily implemented in real-world community settings. BF% and WHR were significantly influenced by increasing age; therefore, our findings support the importance of exercise implementation, especially with increasing age, for the maintenance of a disease-free healthy lifestyle.

## Introduction

Physical inactivity and related health objectives are socially challenging in developed countries and vary according to socioeconomic status [1]. Socioeconomically advantaged people are more likely to be physically active and less likely to have health problems related to sedentary lifestyles than less advantaged populations [2]. Approximately 3.2 million deaths worldwide occur due to physical inactivity [3]. Besides specific suggestions on social health determinants, disparities continue to grow in many countries and are now generally recognized as a problem that requires immediate and significant intervention [4].

Increased participation in physical activity (PA) has become a public health challenge among populations with low socioeconomic status. The significant socioeconomic gradient in PA engagement has been between all age groups, even in early childhood [5]. Moreover, women might be more vulnerable than men because of their gender roles [6]. Most interventions related to PA have been designed and tested in the general population, with less focus on their impact across socioeconomic backgrounds [7, 8]. However, interventions without recognizing the needs and barriers related to participation in PA of the socioeconomically disadvantaged population may seem ineffective for health promotion [9, 10]. The significant sociocultural barriers among the disadvantaged group of women that hinder them from attaining optimal PA are motherhood, domestic tasks, social support, and lack of time [6]. Therefore, effective and time-efficient health-promoting interventions for women in the real-world settings need to be incorporated into their daily routine for maintaining a healthy lifestyle.

Interval-based high-intensity circuit training (HICT) is characterized by a short burst of intense exercise with brief periods of rest [11]. It comprises multiple rounds that alternate between several minutes of high-intensity exercise to increase the heart rate to at least 80% of maximum heart rate (MHR) and a short recovery period. Previous studies have conducted HICT exercise regimens; one of the studies compared high-intensity interval-based training with moderate-volume training. The training intervention was administered for only five weeks, and the population was classified as having class I and II obesity [12]. In another randomized controlled trial, high-intensity interval training (HIIT) and maximal fat oxidation were administered to a male population with obesity, and the BMI was significantly reduced in both groups [13]. A study evaluated the effects of two regimens of HIIT on changes in VO_2max_, body composition, and muscular strength in women with obesity and sedentary lifestyles. The exercise protocol was conducted for six weeks at three sessions per week, in which two sessions were performed in the laboratory setting and one performed outside of the laboratory by the participants themselves. Body fat mass and waist-to-hip ratio (WHR) did not change, although significant changes were observed in body fat percentage (BF%) and fat-free mass [14]. Despite multiple studies being conducted on HICT interventions, data truly representing the effects of HICT on women with overweight, impact of an extended number of exercise sessions, and anthropometric health outcomes on HICT performed outside the laboratory are scarce.

Although ample evidence supports interval-based HICT as an effective strategy for improving health-related markers in laboratory settings, the implementation of HICT in real-world community settings remains a research gap that needs to be considered by researchers, health promoters, and policymakers. Therefore, the aim of the current study was two-fold: first, to implement HICT in the real-world settings, focusing on health promotion among socioeconomically compromised women who are overweight and second, to evaluate the effectiveness of interval-based HICT on anthropometric parameters and identify the relationship between health outcomes including BMI, BF%, and WHR parameters. We hypothesized that the HICT protocol can effectively improve physical fitness, BF%, and WHR among women in low socioeconomic areas who are sedentary and overweight.

## Materials and methods

### Study design and participants

A quasi-experimental study was conducted from January 2021 to November 2021 in the community center park of Ziauddin Hospital in Sikandrabad, a low socioeconomic area. The targeted population was women aged between 18 and 35 years who had a sedentary lifestyle (any resting behavior such as sitting, reclining, or lying position that required ≤1.5 metabolic equivalents of task energy expenditure). Participants were invited through the distribution of flyers within the area with information regarding the free camp exercise service as part of the study. Participants were enrolled using a convenience sampling technique following screening for the study’s inclusion criteria. Women with a BMI of 25.0–29.9 kg/m^2^ and who had a sedentary lifestyle scored ≥26 on the Rapid Assessment Disuse Index questionnaire were included in the study. Meanwhile, those who had any chronic disease (uncontrolled hypertension or diabetes) or history of any abnormal cardiac events; who were pregnant or had history of giving birth in the last 6 months; who actively exercised daily (≥2 days per week); and who answered “yes” to one or more questions on the Physical Activity Readiness Questionnaire for exercise were excluded from the study.

The study sample size was set at 60 participants using the Open Epi software version 3 for a power of 0.80, with an effect size of 0.5 and alpha = 0.05, considering the mean differences and standard deviations of 0.8±1.5 and 2.7±3.1, respectively, with overweight BMI > 25 kg/m^2^.

The study protocol was approved by the Research Ethics Committee of Ziauddin University, Pakistan (ERC No: 2580920SMREH) and the Clinical Trial Protocol Registration and Results System (NCT04655014). The quasi-experimental trial used the Transparent Reporting of Evaluations with Non-randomized Designs checklist and its statement guidelines of 2004 [15, 16]. All interventions and outcome measures were performed in the community’s family park under stable weather conditions and were strictly regulated. Furthermore, the participants were instructed to maintain their usual lifestyle patterns concerning PA and diet to minimize possible confounding effects. Written informed consent was obtained from all participants involved in the study.

### Interventions

A trained physical therapist conducted a six-week HICT; a total of 18 sessions were provided with a frequency of three sessions per week on alternative days. The exercise sessions were performed in a community family park by group; each group had a maximum of ten participants. The duration of each exercise session was 30 min for weeks 1 and 2, based on one set of circuit training performance, which was progressively increased in weeks 3 and 4 to 30–40 min with two sets of circuit training performance and ultimately increased to 40–45 min of exercise performance in weeks 5 and 6 with three sets of circuit training.

Each training session was subdivided into three parts: (i) 10-minute warm-up through brisk walking and easy jogging with an intensity of 50% of MHR; (ii) interval-based HICT at 85%–95% MHR (jumping jacks, wall sits, modified push-ups, abdominal crunches, step up, squats, and planks); and (iii) 10-minute cool-down with deep breathing and slow stretching exercise of major muscles [exercise protocol in the S1 File]. The heart rate was measured at study initiation using a pulse oximeter. During exercise, the participants were instructed to note their exertion level and intensity using the rating of perceived exertion (RPE) scale; the RPE 8–9 scale corresponds to 85% to 95% of the MHR. The MHR was calculated using the following formula: 220 minus the participant’s age.

### Outcome measures

Pre- and post-test measurements were performed at the start of the first session at week 1 and at the end of the last session at week 6. The outcome measures were obtained by another trained therapist who was blinded to the participant’s condition.

Anthropometric readings were considered an outcome measure, including body mass index (BMI), BF%, and WHR. BMI was calculated using weight in kilograms and height in meters squared [17]. BF% was measured using a standardized fat track digital skin fat caliper (AccuFitness, USA). The measurement tool had a 1-mm accuracy and 100% repeatability. The device is considered a reliable tool for measuring subcutaneous fat tissue, with an inter-class coefficient correlation of 0.99 [18]. Body fat calculation is based on the Jackson–Pollock three-site formula, with measurements taken on the triceps, suprailiac, and thigh regions holding the skin from the index finger and thumb to ensure that the skin folds are pinched and not the muscle mass. The average of three consecutive readings was recorded from each site. The caliper recorded three measurements and then automatically calculated and displayed the BF% using the Siri equation with the touch of a button. The WHR was calculated using the following formula: waist circumference/hip circumference. The measurement tape was placed at the midpoint between the last palpable rib and the upper margin of the iliac crest to measure the waist circumference, whereas the circumference of the hip was measured by placing a tape around the widest portion of the buttocks.

### Statistical analysis

Statistical analyses were performed using the IBM SPSS version 20 software. Descriptive statistics were calculated using mean, standard deviation, frequency, and percentage. Normality of the data was analyzed, and skewed data were identified for BMI, BF%, and WHR. The Wilcoxon signed-rank test was used to measure pre- and post-intervention outcomes. Spearman’s correlation coefficients were used to analyze the associations between BMI and BF% and between BMI and WHR. Multiple linear regression and polynomial regression (curve estimation) analyses were performed to identify the relationship and linearity between the dependent and independent variables. Statistical significance was set at P < 0.05.

## Results

**Fig 1:**
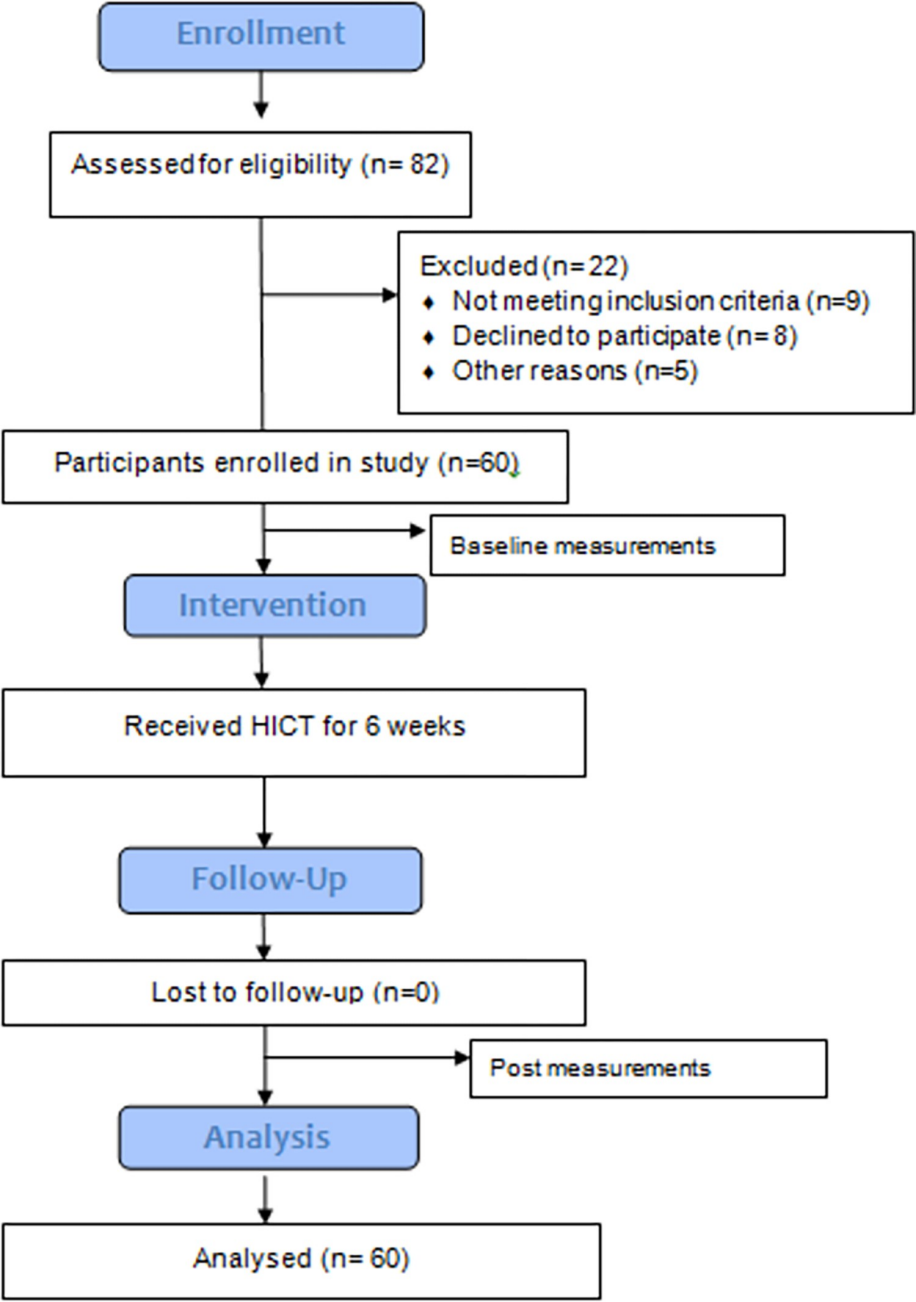
Summary of study participants and selection process.

In total, 60 women completed the training protocol for six weeks from January 2021 to November 2021. The flow chart (Fig 1) provides a summary of the participants assessed for eligibility and those enrolled in the study.

**Fig 2:**
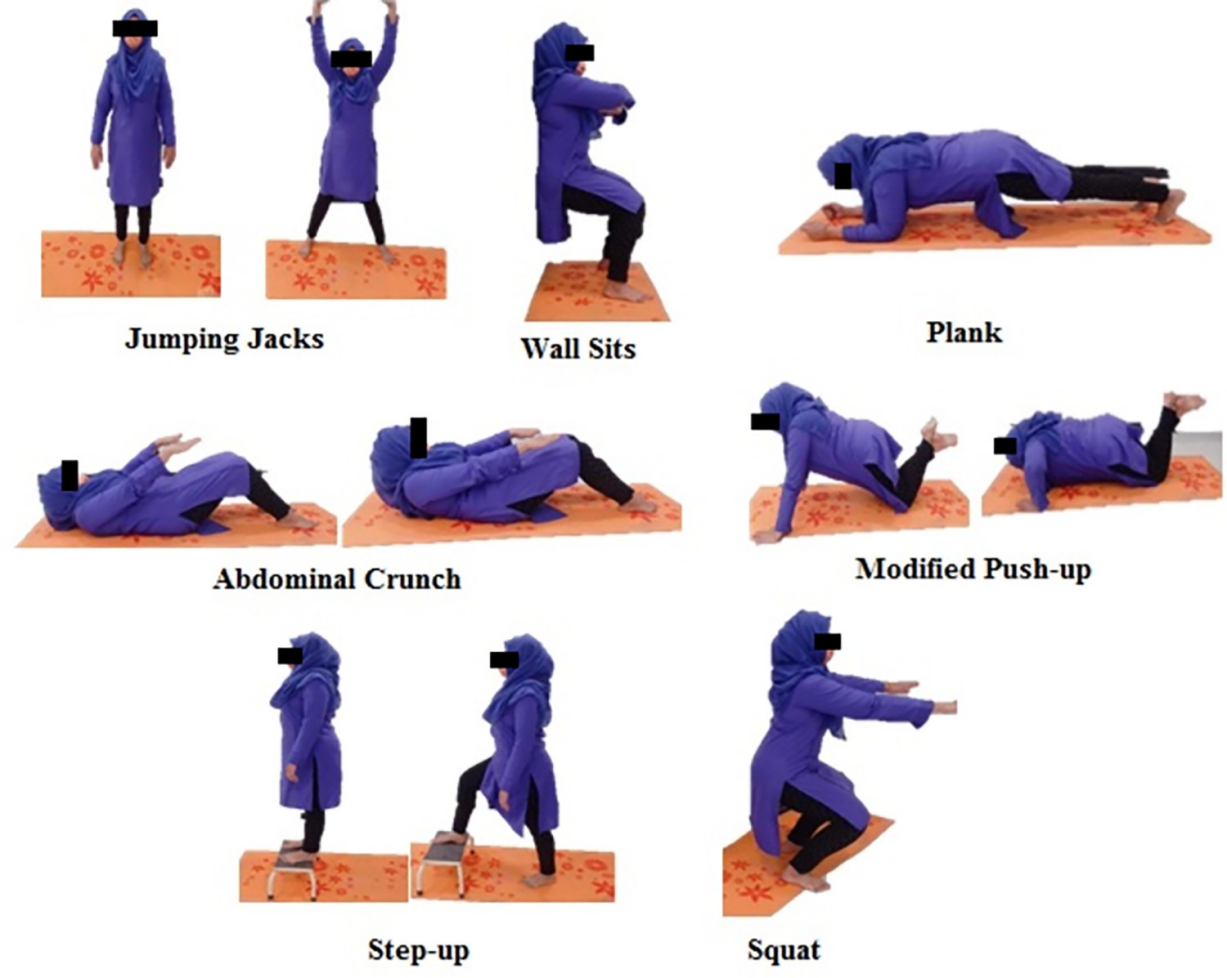
Exercise.

The adherence rate was intended to be the highest (100%) in the daily attendance records of the participants. No adverse events occurred during the training sessions. All participants performed HICT exercises (Fig 2).

**Fig 3:**
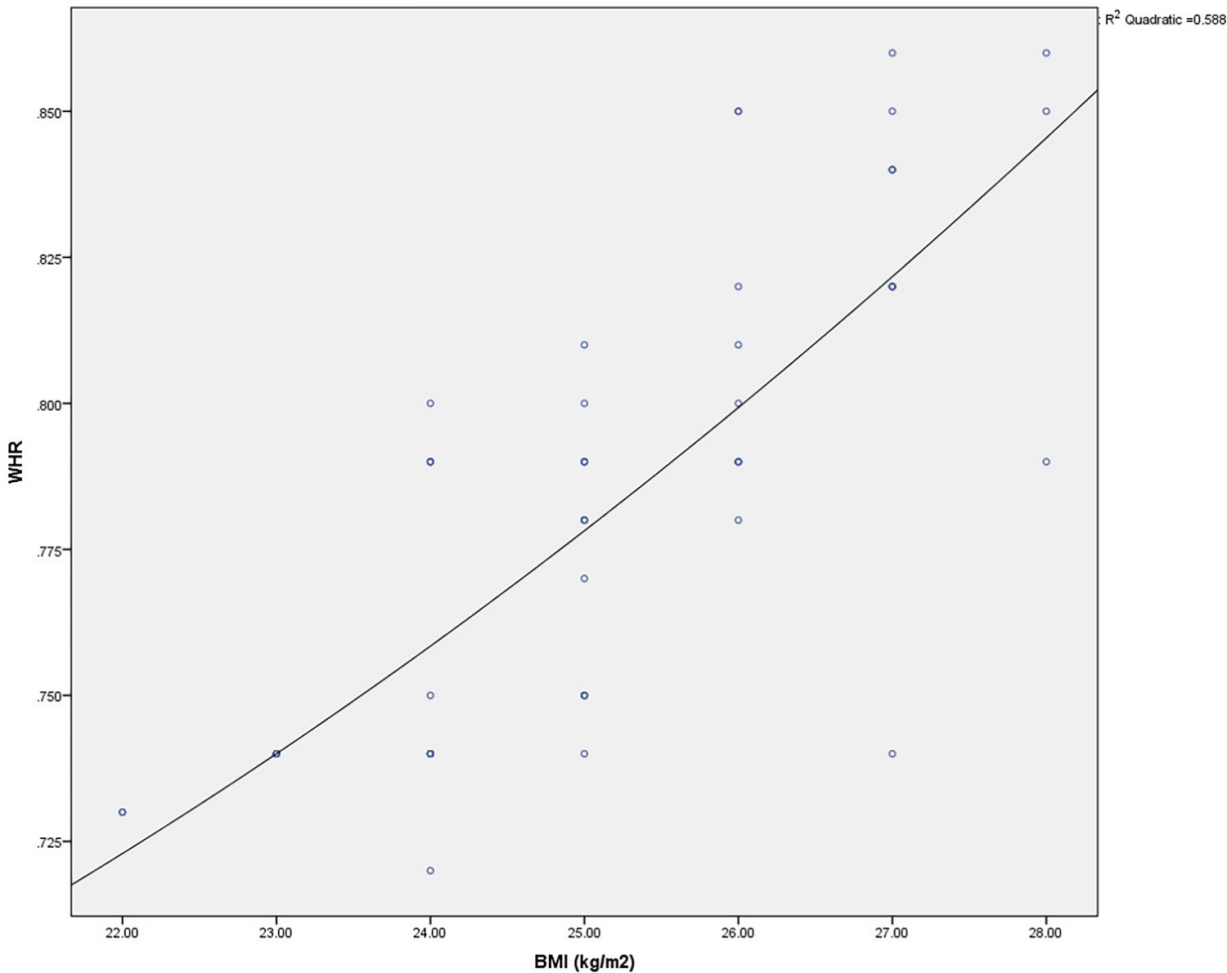
Curve estimation of BMI–WHR relationship using a quadratic model (R^2^ = 0.58, SE = 0.02, *p* <0.001).

**Fig 4:**
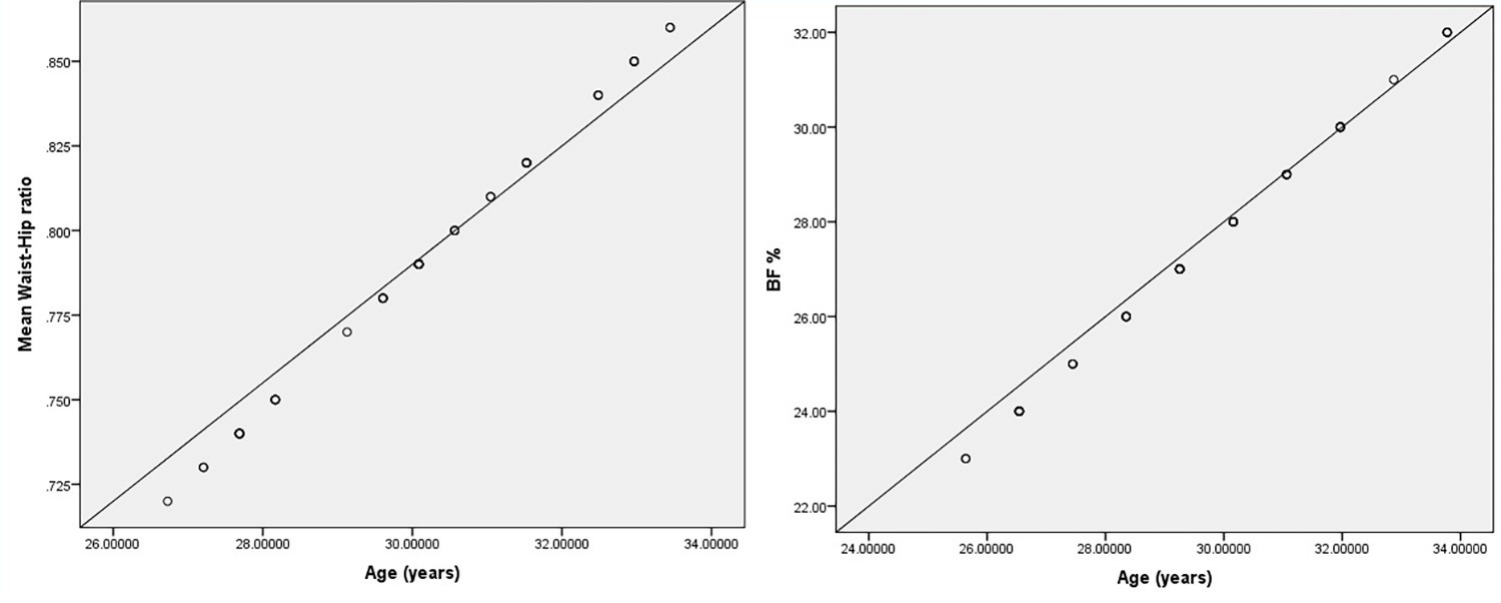
Positive linear relationships between waist-to-hip ratio and age (upper graph) and between body fat percentage and age (lower graph).

The demographic characteristics of the participants are presented in Table 1. Significant differences in BMI (*p*<0.001), BF% (*p*<0.001), and WHR (*p*<0.001) were observed between the pre- and post-interval-based HICT (Table 2). A significant strong correlation was identified between BMI and WHR with a p-value <0.001, while a significant but weak correlation between BMI and BF% was observed (Table 3). Curve estimation using the quadratic model also indicated a positive relationship between BMI and WHR **(**R^2^ = 0.58, SE = 0.02, *p* <0.001) (Fig 3). Multiple linear regression was analyzed, considering age and BMI predictors for BF% and WHR. Age and BMI were significant predictive variables to WHR in the linear regression model (R^2^ = 0.628, F change; 48.1, *p* = <0.001) and BF% (R^2^ = 0.26, F change; 10.06, *p* = <0.001) (Table 4). The scatter plot demonstrated that WHR and BF% linearly increased with age among the young adult women (Fig 4).

**Table 1:** Sociodemographic characteristics of the study participants.

Sociodemographic characteristics	Total (N = 60)
	n (%)
**Marital status**	
Married	46 (76%)
Single	14 (23%)
**Education**	
Primary/secondary school level	58 (96%)
Intermediate level	2 (3.3%)
University level	–
**Age (years)**	Mean±SD
	29.76±4.75

**Table 2:** Pre- and post-reading after 6 weeks of intervention.

Variables	df	Pre-training	Post-training	Mean difference	95% of CI of the difference	*Effect size*	*p*-value
		(mean±SD)	(mean±SD)			*r*	
**BMI**	59	27.3±1.3	25.1±1.4	1.88	1.68 (lower)	0.87	p<0.001
					2.07 (upper)		
**BF %**	59	31.9±2.3	27.6±2.4	4.16	3.88 (lower)	0.87	p<0.001
					4.61 (upper)		
**WHR**	59	0.83±0.04	0.78±0.03	0.79	0.04 (lower)	0.84	p<0.001
					0.05 (upper)		

BMI, body mass index; BF% body fat percentage; WHR, waist-to-hip ratio; Df, degree of freedom; CI, confidence interval.

**Table 3:** Spearman’s correlation of BMI with BF% and WHR.

Independent variable	Dependent variable	*Pearson’s correlation coefficient/r*	*p*-value
**BMI**	WHR	0.76	*p*<0.001
**BMI**	BF%	0.39	0.002

BMI, body mass index; BF%, body fat percentage; WHR, waist-to-hip ratio.

**Table 4:** Multiple regression analysis.

Variable	Predictor	Regression coefficient	SE	β	*F change*	*p*-value
**Waist-to-hip ratio**	Age	0.002	0.001	0.211	*48*.*102*	*p*<0.014
	BMI	0.019	0.002	0.712		*p*<0.001
	R^2^	0.628				*p*<0.001
**Body fat percentage**	Age	0.162	0.196	0.320	*10*.*062*	*p*<0.009
	BMI	0.539	0.059	0.324		*p*<0.008
	R^2^	0.261				*p*<0.001

The variable age was constant. BMI, body mass index; SE, standard error.

## Discussion

This study aimed to determine the practicability and effectiveness of an interval-based HICT protocol among community-dwelling women in a real-world setting. The main findings of this study are as follows: (a) the training protocol resulted in a significant reduction in BMI, BF%, and WHR, reflecting the decreased risk of developing obesity-related diseases; (b) HICT protocols elicited sustained motivation and the adherence rate was high throughout the study, which indicates the feasibility of this exercise regimen among women; and (c) BMI was strongly correlated with WHR and moderately correlated with BF%. However, the WHR and BF% were significantly influenced by increasing age.

Moreover, WHR and BF% increased with age, which supports the importance of exercise in maintaining a healthy lifestyle. Based on the results, the interval-based HICT protocol efficiently and cost-effectively reduces the following anthropometric parameters (BMI, BF%, and WHR) among community-dwelling women. The high adherence rate, motivation, and strict adherence to exercise were assumed to cause the reduction in BMI and BF%. Therefore, promoting PA in socioeconomically disadvantaged populations may be a better approach.

Several studies have demonstrated interval-based HICT as an effective and time-efficient health promotion strategy for improving various health outcomes [19–21]. However, data regarding the feasibility and efficacy of interval-based HICT in real-world settings are limited. Moreover, few studies addressing this issue have reported contradictory results. A study has reported low adherence rates and only slight improvements in cardiorespiratory fitness during a 12-week HICT intervention comprising walking and running protocols in a community park setting [22]. By contrast, Shepherd et al. have reported significant improvements in VO_2max_ after 10 weeks of HICT on indoor spinning bikes in a gym setting as well as greater adherence among HIIT participants compared to MICT participants [23]. Furthermore, two studies have revealed significant increases in VO_2max_ and suggested HIIT as an effective exercise strategy that promotes a healthy lifestyle in a 4-week intervention involving home-based equipment-free high-intensity exercises or a 6-week intense stair climbing intervention [24, 25]. However, these studies used cycling, ergometers, and stair-climbing, and repositories regarding HICT providing body weight as resistance are lacking. However, HICT has recently gained popularity owing to the removal of barriers to accessing tools and facilities when using one’s body weight as resistance. A pilot study of HICT was conducted by Miller et al. [26] using resistance training for 4 weeks, and they identified no significant difference in BMI (p = 0.26), which is contrary to our study, where body weight was used as resistance for 6 weeks, and a significant difference of <0.001 was recorded. However, a similar significant reduction in the BF% was observed (p<0.001). Furthermore, Lee et al. [27] evaluated the effect of a 4-week HICT program among ten inactive women (aged 20–23 years), and the results revealed significant improvement in body weight (P = 0.001), waist circumference (P = 0.003), BMI (P = 0.001), and BF% (P = 0.003). Similar to the previous study, our study observed a significant change in BMI (P = 0.001), WHR (P = 0.001), and BF% (P = 0.001) in a 6-week HICT program despite demonstrating negligible difference in training frequency. Another study conducted in 2015 has reported no significant improvement in BMI (P>0.05) and BF% (P>0.05) among 20 young women with overweight and obesity after a 12-week HICT exercise regimen. However, our study observed significant improvements in BMI and BF% in a 6-week period with a sample size of 60 women with overweight [28]. Ballesta-Garcia et al. [29] assessed the effect of HICT and moderate-intensity circuit training (MICT) twice a week (1 h per session) for 18 weeks among 90 women. They have reported that HICT significantly improved BMI (P = 0.03) compared to MICT (P = 0.14) and concluded that HICT had a better impact in reducing BMI. Comparatively, our study also demonstrated a significant improvement in BMI, with the limitation of the no comparison group.

Our findings support recent research and strongly recommend the use of HICT in real-world fitness routines. Here, the participants were enrolled through community-based networks with an excellent adherence rate recorded at 100%. Bauman et al. have reported that the leading cause of insufficient PA was a "perceived lack of time" [30]. In the present study, most participants perceived that circuit-based HICT intervention was feasible for overcoming prolonged engagement in regular exercise. Therefore, the low-volume HICT exercise strategy in a real-world setting may contribute to decreasing time-related barriers by increasing PA among sedentary individuals.

Moreover, no harmful events occurred during the current study, indicating that HICT can be safely performed in previously undertrained populations in real-world settings. Previously, a meta-analysis was conducted by Weston et al. [31] in which ten out of six studies implemented 85% to 95% of HIIT similar to that in our research; among them, one study was conducted in adults with obesity [32]. However, they monitored the intensity of MHR using heart rate as a laboratory-based intervention; further, when home sessions were taught in that study, participants were instructed to use the RPE scale. Correspondingly, as our research aims to provide intervention in a real-world setting, instead of laboratory-based interventions, we used the RPE scale to monitor the intensity. The HIIT was better than MICT in decreasing weight and improving aerobic work capacity. To the best of our knowledge, evidence for determining the effects of low-volume circuit-based HICT on anthropometric parameters among community-dwelling sedentary women is limited. Our results indicate that HICT can significantly reduce BMI, BF%, and WHR; these findings are relevant for health professionals to implement an effective strategy in community settings. Further studies are needed to examine the feasibility and effectiveness of interval-based HICT interventions in community settings in a larger population.

Our study had a few limitations, including the non-availability of a control group and the lack of strict monitoring of either diet consumption or PA outside the study. Strict observation of these factors is highly recommended for future studies. Our study was confined to a single under-privileged community that should be generalized to a multi-community population for further studies. Further studies should be conducted that consider other categories of obesity.

## Conclusion

A positive correlation was identified between BMI-WHR and BMI-BF% among young adult community-dwelling women. Therefore, our findings support the importance of considering BMI when predicting BF% and WHR in individuals with overweight. Moreover, WHR and BF% were significantly influenced by age; therefore, our findings support the importance of exercise, especially with increasing age, for maintaining a healthy disease-free lifestyle. Furthermore, our data suggest that equipment-free interval-based HICT can easily be implemented in real-world community settings, which require little time commitment and may reduce BMI, BF%, and WHR.

## Supporting information

S1 Checklist (DOCX)

S1 File (DOCX)

S2 File (PDF)
