# Supplementary material for: High-intensity circuit training for improving anthropometric parameters for women from low socioeconomic communities of Sikandarabad: A clinical trial
Source: PLoS One. 2022 Oct 17;17(10):e0275895. doi: 10.1371/journal.pone.0275895 (PMC9576086; doi:10.1371/journal.pone.0275895)
Supplement: S1 File — (DOCX) [file pone.0275895.s002.docx]

## Training protocol:

**High Intensity Circuit Training:**

The High-Intensity Circuit Training comprises of 7 full-body exercises to involve the major muscle groups of an individual. As per the recommendation of ACSM each participant attended a total of 18 sessions of their HICT protocol, comprised of High Intensity circuit training, 3 times/ week for six weeks.

All participants performed the High-intensity circuit training protocol as per ACSM guidelines; FITT's protocol (Frequency, Intensity, Time, and Type).

**Frequency:** 3 days/week for 6 weeks

**Intensity:** 85-95% of Maximum Heart Rate (MHR).

**Time:** 30-45 minutes

**Type:** High intensity Interval training using body weight as a resistance

The participants were advised to wear comfortable clothing during exercise sessions. Hence, HICT protocol included a total number of seven stations with major muscle group physical activity involving Jumping jacks, wall sits, modified pushups, abdominal crunches, step-up, squats, and planks. Each session of exercises was initiated from warm-up activity progress to circuit-style training and ended up with a cool-down session respectively.

In the first two weeks, the entire circuit was performed only once, in the second and third week; two sets of circuits were performed whereas in the fifth and sixth week three sets of circuit were performed in a similar manner. HICT was performed in the following order for six weeks of duration.

## Warm-up

Five to ten minutes warm-up exercises were performed before initiation of the exercise protocol that comprises of brisk walking and easy jogging with an intensity to increase +10 beats in Resting Heart Rate (RHR) measured by a pulse oximeter. The purpose of the warm-up is to increase body temperature by raising the heart rate 10 beats above resting level.

## High intensity circuit training

1. **Jumping Jacks**: is a side-saddle hop, subjects performed jumping with the leg spread wide apart and hand claps overhead with returning to a position with feet together and arm aside, jumping jacks were performed for 30 seconds followed by 30 seconds period of rest.
2. **Wall sits:** The subjects placed their back against the wall with hip and knee in right angle by holding the position for 30 seconds in the given time, followed by a rest period of 30 seconds
3. **Modified Pushups:** The individual adopted prone position and shift their weight on both knees and hands; exercise was performed by rising and lowering the upper body with fixed hands and knees on floor, this activity was performed continuously for 30 second followed by 30 seconds period of rest.
4. **Abdominal crunches**: subject adopted lying position by facing upward, both knees were bending on 90 degrees and both hands werewrapped behind the individual’s head, exercise was performed by raising their upper body towards the knees without moving the legs. Abdominal crunches were performed for 30 seconds followed by 30 seconds period of rest.
5. **Step up**: a 16 inches stepper was placed in front of individual, exercise was performed by stepping up with one leg followed by stepping down with same leg and repeated this activity with other leg. Both legs were engaged in this exercise for continuous 30 seconds followed by 30 seconds of rest period.
6. **Squats:** The movement began while standing straight and both hands in front reach position, exercise was perform by descending down their torso with straight spine by bending both knees together. Squatting was performed for continuous 30 seconds followed by 30 seconds of rest period.
7. **Planks:** The subject was asked to lie on prone position and raising their whole body upward and shift their weight on both toes and elbows by holding the position for 30 seconds. If individual was failing to achieve this position for 30 seconds she was asked to hold the position of plank as long as she can.


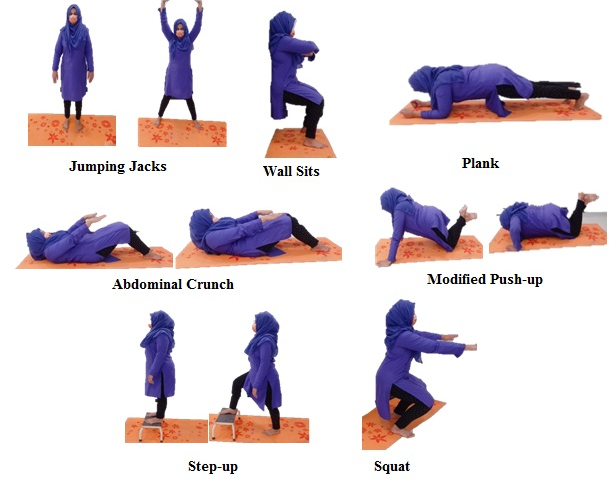


## Exercise termination criteria

Exercise will be terminated on the happening of any one of the following events

- Decrease in oxygen saturation < 90%assessed through a pulse oximeter.
- Signs of poor perfusion (circulation or blood flow), including pallor (pale appearance to the skin), cyanosis (bluish discoloration), or cold and clammy skin
- Increase in Heart Rate above the highest limits of Targeted Heart Rate (THR) calculated by Karvonen formula.
- The exercise shall be terminated immediately if subject feel any discomforts, dizziness, fainting or difficulty in breathing during performance.
- The exercise shall be terminated upon request of the participants.
