# Supplementary material for: High-intensity circuit training for improving anthropometric parameters for women from low socioeconomic communities of Sikandarabad: A clinical trial
Source: PLoS One. 2022 Oct 17;17(10):e0275895. doi: 10.1371/journal.pone.0275895 (PMC9576086; doi:10.1371/journal.pone.0275895)
Supplement: S2 File — (PDF) [file pone.0275895.s003.pdf]

STUDY PROTOCOL

RESEARCH PROPOSAL

"Effects of HighIntensity Circuit Training on PhysicalFitness, Body Fat Percentage and Waist-Hip Ratio among Sedentary Females of Sikandarabad"

INTRODUCTION

Globally, insufficient Physical Activity (PA) and sedentary life style is recognized as major causes of mortality among both young and older adults (Ozemek et al., 2019). Approximately, 5 million deaths are attributed due to physical inactivity (PI), which contributes around 6% of global death (Rawal et al., 2020). The recent evidence shows that 80 million individuals in Pakistan suffered with non-communicable diseases, due to PI that ultimately lead to sedentary behavior (Memon et al., 2020). As per the data documented by World Health Organization, Global Health Observatory (GHO) in 2016, almost 28% of populations 18+ years were found to be PI. It was therefore estimates that this PI may lead to dyslipidemia, hypertension and obesity. Therefore, the clinical practitioners have been highlighting in improving the physical activity among young adults not only to prevent chronic disease but also to induce healthy behaviours (Vlaar et al., 2017). Despite of the fact, a large number of young adults are PI where this proportion remains high among women (Sloutskis et al., 2010, Kahan et al., 2017). Several studies have documented that the accessibility to perform PA differs in different socio-demographic communities especially among low socioeconomic status (SES) (Stalsberg et al., 2018, Moore et al., 2015). Moreover, it has also been reported that individuals with low SES have less time to participate in organizing leisure time physical activity, than high SES (Stalsberg et al., 2018) mostly due to cost.

WHO reports that Generally, PI is found to be more prevalent in women, due to workload of house chores and care giving role in influence of cultural expectations especially in low SES. Furthermore, several other factors including high cost of fitness programs, work schedule, transportation and most importantly time barrier are the major cause of insufficient PA (Argent et al., 2018).Addressing the above barriers, different exercise training protocols have been developed to reduce weight in short span of time and then to maintain it. According to American College of Sports Medicine (ACSM), 150 minutes/week of moderate intensity of exercise is recommended for older adult population. With the advent of time, ACSM recommended combination therapies including High Intensity Circuit Training (HICT) because of increased efficiency and practicality. The concept is to combine the aerobic and resistance training into one bout of approximately 7 minutes and then repeating the bout 2 to 3 times progressively (Klika et al., 2013). HICT is a circuit-style workout that uses individual body weight as a resistance (Clayton et al., 2019). therefore eliminating the need of expensive gym equipment such as dumbbells, barbells kettle bells and many more. In addition, HICT can be performed in any environment (at home, parks and in small place) as per the feasibility of the person Even the number of exercise stations can vary with multiple large muscle groups and completing the bout of 30 seconds with very short rest period between exercise stations (Klika et al., 2013). On the other hand, a randomized control trial reported that overweight and sedentary women are found to adhere easily with short bout of exercises due to providing access of home exercise equipments for improving long term weight loss , (Jakicic et al., 2017). Although ample evidence supports interval-based HICT as an effective strategy for improving health-related markers in laboratory settings, the implementation of HICT in real-world community settings remains a research gap that needs to be considered by researchers, health promoters, and policymakers.

Therefore, the aim of this study is to implement HICT in the real-world settings, focusing on health promotion among socioeconomically compromised women who are overweight and secondly to provide cost effective and efficient exercise intervention that may improve anthropometric parameters.

STATEMENT OF THE PROBLEM

Global burden of obesity is increasing with the passage of time due to PI and sedentary lifestyle that has been adopted by women in the society owing to time constraints as well as increased cost; ultimately decreasing the quality of life among young adults of low socioeconomic class. Therefore, a dire need has been arise by the clinicians to cater this problem in a time effective way that is easily adopted and implemented to physical fitness and body composition for healthy lifestyle.

RESEARCH QUESTION

Is High Intensity Circuit training effective in improving the anthropometric parameters among overweight females of low socioeconomic area?

OBJECTIVES

- To assess the effects of High intensity circuit training on body mass index among overweight sedentary females of low-socioeconomic areafollowing six weeks of training.
- To assess the effects of High intensity circuit training on body fat percentage among overweight sedentary females of low-socioeconomic area following six weeks of training.
- To assess the effects of High intensity circuit training on waist-hip ratio among overweight sedentary females of low-socioeconomic area following six weeks of training.

HYPOTHESIS

H_A: The High Intensity Circuit Training protocol is effective in improving body mass index, body fat percentage and Waist-Hip ratio among sedentary overweight females of low socioeconomic area.

RATIONALE

HICT has been growing in popularity because of its efficiency and practicality with numerous health benefits in less time consumption as well as cost effectiveness. Improving the PA as per the ACSM guidelines among young adults shall not only decrease the global burden of non-communicable disease but also improves the later on risk factors associated with it. Although internationally, limited evidence is available considering the circuit training programs for young adults but nationally to the knowledge of author no data is available yet that deals with it. Therefore, this raises the need to initiate a program that caters the physical fitness of low socioeconomic community of our country.

METHODOLOGY

Study Setting: Study will be conducted at community center park of Ziauddin Hospital in Sikandrabad

Target Population: Overweight young adult females of Sikandarabad community, aged 18-35 years old.

Study Design: Quasi-experimental study design

Duration of Study: 10 to 12 months following the approval of synopsis.

Sample Size: The sample size of 60 is calculated through Open EPI software, an open source calculator by using a reference study conducted Germany in the year 2016 titled as "*Functional High Intensity Circuit Training improves body composition, peak oxygen uptake, strength and alter certain dimension of quality of life in overweight women*", considering 95% of confidence Interval with 80% Power, Mean Differences and Standard Deviations of 0.8±1.5 and 2.7±3.1, respectively.

Sample Selection

Inclusion Criteria

- Female with age in between 18-35 years old.
- Overweight females with BMI in between 25-29 kg/m2
- Having a sedentary lifestyle, ≥26 score on the Rapid Assessment Disuse Index questionnaire

Exclusion Criteria

- Recent surgeries or trauma hinder participation in high-impact exercises: diagnose cases of severe orthopedic, musculoskeletal, cardiopulmonary, neurovascular, psychiatric, inflammatory, metabolic, or endocrine diseases.
- Females are taking any prescribed medications of chronic disease
- Females who actively exercised daily (≥2 days per week)
- Participants who are pregnant, or given birth in the last six months
- Individuals who answered "yes" to one or more questions on the Physical Activity Readiness Questionnaire (PAR-Q & YOU).

Data Collection Procedure:

A total of sixty overweight females will be invited to participate in the study after fulfilling the inclusion criteria. A screening camp shall be arranged two days before enrolling in the program where they shall be screened using BMI, Rapid Assessment Disuse Index questionnaire and Physical Activity Readiness Questionnaire (PAR-Q & YOU) prior to participation. The subjects will be verbally informed about all procedures and protocol and if willing to participate, a written consent along with participation information sheet shall be taken from the participants ensuring that they are well aware of the circuit training protocol. After screening, participant will be allowed to attend the orientation session, where a qualified and trained instructor shall discuss and describe the exercise training protocol. In order to improve the compliance and motivate the participants a WhatsApp group shall be created for reminder. A day prior to initiate the training protocol, anthropometric measurements including weight, height, body fat percentage and waist-hip-ratiowill be measured. The participants will be arranged in groups, maximum of ten

participants in each group and they will complete a six-week HICT; a total of 18 sessions with a frequency of three sessions per week on alternative days. The training protocol will be given as per the recommendation of ACSM standardized guidelines (Klika et al., 2013)

Intervention/Training Protocol:

High Intensity Circuit Training:

The High-Intensity Circuit Training comprises of 7 full-body exercises to involve the major muscle groups of an individual. As per the recommendation of ACSM each participant will attend a total of 18 sessions of HICT protocol, comprised of High Intensity circuit training, 3 times/ week for six weeks.

All participants will perform the High-intensity circuit training protocol as per ACSM guidelines; FITT's protocol (Frequency, Intensity, Time, and Type).

Frequency: 3 days/week for 6 weeks

Intensity: 85-95% of Maximum Heart Rate (MHR).

Time: 30-45 minutes

Type: High intensity Interval training using body weight as a resistance

The participants will be advised to wear comfortable clothing during exercise sessions. Hence, HICT protocol include a total number of seven stations with major muscle group physical activity involving Jumping jacks, wall sits, modified pushups, abdominal crunches, step-up, squats, and planks illustrated in figure 1. Each session of exercises will initiate from warm-up activity progress to circuit training and ended up with a cool-down session respectively.

In the first two weeks, the entire circuit will be performed only once, in the second and third week; two sets of circuits will be performed whereas in the fifth and sixth week three sets of circuit will be performed in a similar manner. HICT will be performed in the following order for six weeks of duration.

Warm-up

Five to ten minutes warm-up exercises willperform before initiation of the exercise protocol that comprises of brisk walking and easy jogging with an intensity to increase +10 beats in Resting Heart Rate (RHR) and will measured by a pulse oximeter. The purpose of the warm-up is to increase body temperature by raising the heart rate 10 beats above resting level.

High intensity circuit training

1. **Jumping Jacks**: is a side-saddle hop, subjects will perform jumping with the leg spread wide apart and hand claps overhead with returning to a position with feet together and arm aside, it will perform for 30 seconds followed by 30 seconds period of rest.
2. **Wall sits:** The subjects will place their back against the wall with hip and knee in right angle by holding the position for 30 seconds in the given time, followed by a rest period of 30 seconds
3. **Modified Pushups:** The individual will adopt prone position and shift their weight on both knees and hands; exercise will perform by rising and lowering the upper body with fixed hands and knees on floor, this activity will perform continuously for 30 second followed by 30 seconds period of rest.
4. **Abdominal crunches**: Subject will adopt lying position by facing upward, both knees will bend on 90 degrees and both hands will be wrapped behind the individual’s head, exercise will perform by raising their upper body towards the knees without moving the

legs. Abdominal crunches will be performed for 30 seconds followed by 30 seconds period of rest.

5. **Step up**: A 16 inches stepper will be placed in front of individual, exercise will be performed by stepping up with one leg followed by stepping down with same leg and will ask to repeat this activity with other leg. Both legs will engage in this exercise for continuous 30 seconds followed by 30 seconds of rest period.
6. **Squats:** The movement began while standing straight and both hands in front reach position, exercise will perform by descending down their torso with straight spine by bending both knees together. Squatting will perform for continuous 30 seconds followed by 30 seconds of rest period.
7. **Planks:** The subject will asked to lie on prone position and raise their whole body upward and shift their weight on both toes and elbows by holding the position for 30 seconds. If individual will fail to achieve this position for 30 seconds she will be asked to hold the position of plank as long as she can.

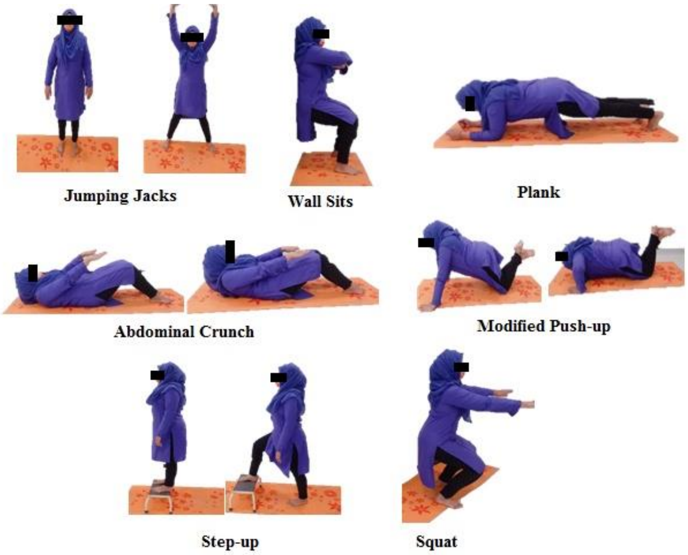

Figure 1: Exercise

Cool Down

Cool down session shall comprise deep breathing exercises and slow stretching exercise of major muscles for up to ten minutes.

Exercise termination criteria

Exercise will be terminated on the happening of any one of the following events

- Decrease in oxygen saturation < 90%assessed through a pulse oximeter.
- Signs of poor perfusion (circulation or blood flow), including pallor (pale appearance to the skin), cyanosis (bluish discoloration), or cold and clammy skin
- Increase in Heart Rate above the highest limits of Targeted Heart Rate (THR) calculated by Karvonen formula.
- The exercise shall be terminated immediately if subject feel any discomforts, dizziness, fainting or difficulty in breathing during performance.
- The exercise shall be terminated upon request of the participants.

Outcome measures:

All outcome measure will be taken before the start of first session and after six week of exercise session. The outcome measures will be obtained by another trained therapist who will be blinded to the participant's condition. Anthropometric parameters will be considered as an outcome measure, including body mass index, body fat percentage and waist hip ratio.

- **Body mass index:**

  BMI will calculate by formula:

  BMI = weight ÷ $height^2$

  The weight in kilograms and height in meters squared will measure using stadiometer.

- **Body fat percentage:**

  Body fat percentage will measure through a standardized fat track digital skin fat caliper.The device is considered a reliable tool for measuring subcutaneous fat tissue, with an inter-class coefficient correlation of 0.99. (Siatras et al., 2010) Measurements will be taken on the triceps, suprailiac, and thigh region by holding the skin from the index finger and thumb to ensure that the skin folds are pinched and not the muscle mass. The average of three consecutive readings will be recorded from each site. The caliper will record three measurements and then automatically calculate and displayed the bf% with the touch of a button.

- **Waist-hip ratio:**

  The WHR will calculate using the formula:

  Waist circumference/hip circumference.

  The measurement tape will placeat the midpoint between the last palpable rib and the upper margin of the iliac crest to measure the waist circumference, whereas the circumference of the hip will measure by placing a tape around the widest portion of the buttocks.

Data Analysis Procedure:

Data will be analyzed by using SPSS version 22.0.

The demographic information will be represented using descriptive statistics such as frequency, percentage, mean and standard deviation. The data will be analyzed for normality using skewness and kurtosis method. If the data is found to be normally distributed then for inferential statistics, paired T-Test will be run whereas if the data is not found to be normally distributed

then Wilcoxon Signed-Rank test will be applied. The level of significance p value < 0.05 will be considered significant.

Ethical Considerations

The study will be performed according to the deceleration of Helenski. The participants data will be kept confidential and all the participants will have right to withdraw study at any time. The individual pictured in Figure 1 has provided written informed consent (as outlined in PLOS consent form) to publish their image alongside the manuscript.

REFERNCES:

Clayton, B.C., Tinius, R.A., Winchester, L.J., Menke, B.R., Reece, M.C. and Maples, J.M., 2019. Physiological and perceptual responses to high-intensity circuit training using body weight as resistance: Are there sex-specific differences?. *International Journal of Exercise Science*, *12*(4), p.245.

Jakicic, J.M., Winters, C., Lang, W. and Wing, R.R., 1999. Effects of intermittent exercise and use of home exercise equipment on adherence, weight loss, and fitness in overweight women: a randomized trial. *Jama*, *282*(16), pp.1554-1560.

Kahan, D., 2015. Adult physical inactivity prevalence in the Muslim world: Analysis of 38 countries. *Preventive medicine reports*, *2*, pp.71-75.

Klika, B. and Jordan, C., 2013. High-intensity circuit training using body weight: Maximum results with minimal investment. *ACSM's Health & Fitness Journal*, *17*(3), pp.8-13.

Memon, A.R., 2020. Physical activity to prevent non-communicable diseases: current status and national-level policy in Pakistan. *Journal of Pakistan Medical Association*.

Moore, G.F. and Littlecott, H.J., 2015. School-and family-level socioeconomic status and health behaviors: multilevel analysis of a national survey in Wales, United Kingdom. *Journal of School Health*, *85*(4), pp.267-275.

Ozemek, C., Lavie, C.J. and Rognmo, Ø., 2019. Global physical activity levels-Need for intervention. *Progress in cardiovascular diseases*, *62*(2), pp.102-107.

Rawal, L.B., Smith, B.J., Quach, H. and Renzaho, A., 2020. Physical Activity among Adults with Low Socioeconomic Status Living in Industrialized Countries: A Meta-Ethnographic Approach to Understanding Socioecological Complexities. *Journal of Environmental and Public Health*, *2020*.

Siatras T, Skaperda M, Mameletzi D. Reliability of anthropometric measurements in young male and female artistic gymnasts. Med Probl Perform Art. 2010; 25: 162-166.

Stalsberg, R. and Pedersen, A.V., 2018. Are differences in physical activity across socioeconomic groups associated with choice of physical activity variables to report?. *International journal of environmental research and public health*, *15*(5), p.922.

Vlaar, E.M., Nierkens, V., Nicolaou, M., Middelkoop, B.J., Busschers, W.B., Stronks, K. and van Valkengoed, I.G., 2017. Effectiveness of a targeted lifestyle intervention in primary care on diet and physical activity among South Asians at risk for diabetes: 2-year results of a randomised controlled trial in the Netherlands. *BMJ open*, *7*(6), p.e012221.

Zimmermann-Sloutskis, D., Wanner, M., Zimmermann, E. and Martin, B.W., 2010. Physical activity levels and determinants of change in young adults: a longitudinal panel study. *International Journal of Behavioral Nutrition and Physical Activity*, *7*(1), pp.1-13.

ANNEXURES:

WAIST-HIP RATIO INTERPRETATION SCALE

WAIST-HIP RATIO				
Gender	Excellent	Good	Average	At Risk
Male	<0.85	0.85-0.89	0.90-0.95	>0.95
Female	<0.75	0.75-0.79	0.80-0.86	>0.86

BODY FAT PERCENTAGEINTERPRETATION SCALE

BODY FAT PERCENTAGE IN WOMEN				
Age	Lean	Ideal	Average	Over Fat
Up to 20	11-18%	18-23%	23-30%	30-35%
21-25	12-19%	19-24%	24-30%	30-35%
26-30	13-20%	21-25%	25-31%	31-36%
31-35	13-21%	21-26%	26-33%	33-36%
36-40	14-22%	22-27%	27-34%	34-37%
41-45	14-23%	23-28%	28-35%	35-38%
46-50	15-24%	24-30%	30-36%	36-38%
51-55	16-26%	26-31%	31-36%	36-39%
56 & Up	16-27%	27-32%	32-37%	37-40%
